# Research on Improved MOF Materials Modified by Functional Groups for Purification of Water

**DOI:** 10.3390/molecules28052141

**Published:** 2023-02-24

**Authors:** Junyan Liu, Yang Wang

**Affiliations:** School of Chemistry and Chemical Engineering, Yangzhou University, Yangzhou 225002, China

**Keywords:** MOFs, functional groups, pollutants, water purification, application

## Abstract

With the rapid development of urbanization and industrialization, water contamination has gradually become a big problem. Relevant studies show that adsorption is an efficient strategy to treat pollutants in water. MOFs are a class of porous materials with a three-dimensional frame structure shaped by the self-assembly of metal centers and organic ligands. Because of its unique performance advantages, it has become a promising adsorbent. At present, single MOFs cannot meet the needs, but the introduction of familiar functional groups on MOFs can promote the adsorption performance of MOFs on the target. In this review, the main advantages, adsorption mechanism, and specific applications of various functional MOF adsorbents for pollutants in water are reviewed. At the end of the article, we summarize and discuss the future development direction.

## 1. Introduction

After a protracted battle, humans have triumphantly changed the course of nature and created social economics. In the meantime, environmental destruction has posed a realistic threat to mankind’s existence and progression. Water is the source of life. Oceans cover about 70 percent of the Earth. However, the freshwater resources that can be directly used by people contain less than 1% of the total water in the world. It is worth noting that the quality of available freshwater resources for humans is deteriorating due to demographic and environmental factors. [1,2,3]. At present, every year in some poor and backward countries, about 2 million people suffer from related diseases and eventually die because of a lack of access to clean water [4].

In water, most of the pollutants can be divided into heavy metal ions, acid ions, organic dyes, organic poisons, and drugs, which, to a certain extent, will cause allergy symptoms, nausea, headaches, heart disease, and other symptoms, and even lead to death [5]. A number of strategies have been used to treat these pollutants. They include nanofiltration [6], solvent extraction [7,8], resin use [9], photocatalytic degradation [10,11], cooling, ion exchange, chemical precipitation, electrochemical deposition, reverse osmosis, biological treatment [12,13,14,15], and others. However, most of these technologies have certain shortcomings, such as expensive equipment, complicated operations, and a long experimental period. Fortunately, adsorption, as one of the most promising pollutant treatment methods, has attracted the research interest of many researchers.

For the sake of adsorbing pollutants in water, it is necessary to choose the right material. Metal-organic frame materials were first proposed in 1999 by Omar Yaghi and his team at the University of California, Berkeley [16]. The material has the advantages of low density, a large specific surface area [17], ideal porosity [18,19], big pore size, while being adjustable [20,21,22,23]. Therefore, it presents an attractive topic in the fields of catalytic conversion [24,25,26,27], gas storage [28,29,30], electrochemical biosensing [31,32,33], drug slow-release control [34], molecular recognition [35,36,37], magnetism [38], adsorption and separation of pollutants [39,40,41], and so on. In order to fully exploit the advantages of MOFs in water purification, researchers modified the functional groups on MOFs so as to further boost the adsorption performance of MOFs. All these MOFs have achieved remarkable results in water purification.

## 2. Several Advantages of MOFs Modified by Functional Groups as Adsorbents

The pristine MOFs have some disadvantages, such as low catalytic activity, electrical conductivity, and stability. These shortcomings have limited their achievements in water purification to some extent. These properties are enhanced by modifying the functional groups. However, the most attractive aspect is that they maintain several advantages of the pristine MOFs, which are crucial for the design of different MOFs for water purification and the removal of pollutants from water.

### 2.1. Large Specific Surface Area

Specific surface area means the overall area of the material per unit mass. One of the crucial characteristics of MOFs is their huge specific surface area. The larger the specific surface area, the better the surface properties (such as catalytic capacity, surface adsorption capacity, and surface activity) of the material. For example, Prof. Omar Yaghi et al., the founders of MOF materials, synthesized MOF-5 with a simple cubic structure using terephthalic acid (PTA) as the organic ligand and Zn as the metal center [42]. The material has a large surface area. By calculating the experimental data, we can work out that the specific surface area of MOF-5 is 2900 m^2^·g^−1^. After that, no matter what method researchers used to synthesize MOF-5, their average surface area was greater than 2000 m^2^/g. In 2004–2005, Gerard Ferey’s research group successively synthesized MIL-100 [43] and MIL-101 [44] by using Cr as the metal center and using 1,3,5-benzenetricarboxylic acid (BTC) and 1,2-benzenedicarboxylic acid (BDC) as ligands. Their specific surface areas are 3100 m^2^·g^−1^ and 5900 m^2^·g^−1^, accordingly. Their work can be considered to have opened a new chapter in the development of MOF materials. Now, some studies have shown that the modification of MOFs with appropriate functional groups is beneficial to the increase of specific surface area to a certain extent.

### 2.2. Porous and Adjustable Structure

**Figure 1:**
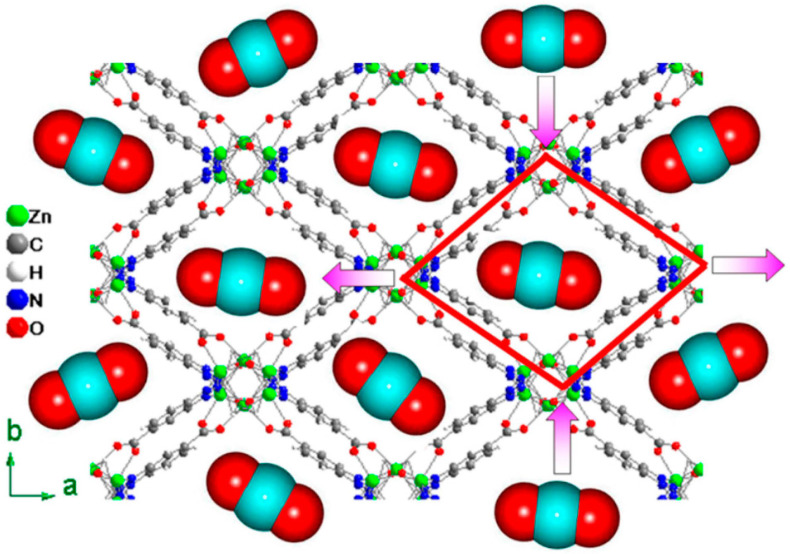
Selective adsorption of CO_2_ by [Zn_3_(btca)_2_(OH)_2_](guest)_n_. Reprinted with permission from Ref. [53]. Copyright 2015, American Chemical Society.

A pore is a hollow area in a porous substance where guest molecules have been eliminated. For MOFs, there are many openings on the surface of functional materials. These pores are essential for the use of MOFs. This feature enables MOFs to be successfully applied to the storage of small molecular gases (H_2_ [45], CO_2_ [46], CH_4_ [47], H_2_O [48], etc.), gas separation (H_2_/CO_2_ [49], CO_2_/N_2_ [50], CO_2_/CH_4_ [51], C_3_H_6_/C_3_H_8_ [52], etc.), and pollutant adsorption. By utilizing various metal centers and ligands, we can modify the structure of MOF materials to produce a range of materials. Yue et al. synthesized [Zn_3_(btca)_2_(OH)_2_](guest)_n_ materials, for example [53]. Taking advantage of the framework’s flexibility, this material can be highly adsorbed with CO_2_ in the presence of N_2_, He, and Ar. This example shows that MOFs are both porous and flexible (Figure 1). MOFs with modified functional groups can still maintain this property. There are still openings on the surface of the material, and different ligands can be selected to adjust the structure and modify different functional groups.

### 2.3. Coordinated Unsaturated Metal Sites

**Figure 2:**
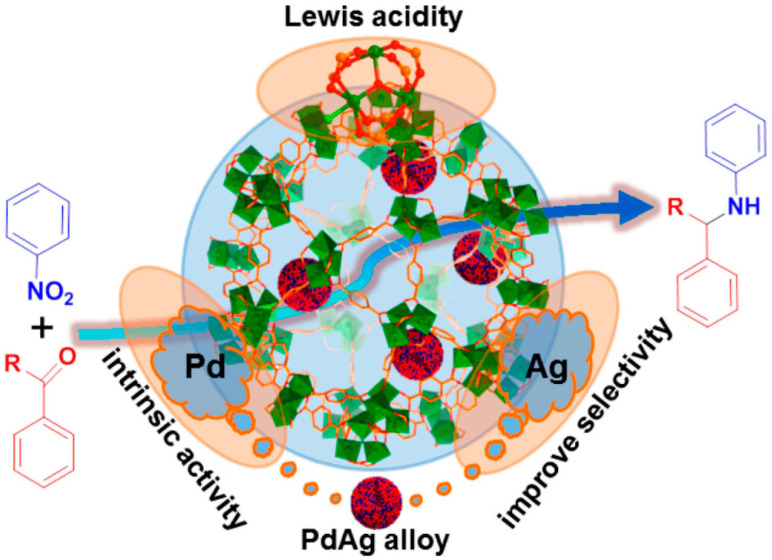
Schematic illustration showing the multi-step reaction over PdAg@MIL-101 involving Pd/Ag sites and Lewis acid. Reprinted with permission from Ref. [55]. Copyright 2015, American Chemical Society.

A coordinated unsaturated metal site is one where the outer orbitals of the metal site are not filled with electrons. We can also understand them as coordinated open metal sites. Unlike noble gases, it does not achieve an electron-stable structure [54]. MOFs contain abundant unsaturated coordination metal sites, which are not only easy to exchange but also can act as the lone pair electron interaction between Lewis acid and the oxygen atom in the C=O double bond, thus reducing the bond energy of the C=O double bond and promoting the shaping of unsaturated alcohols. Chen et al. synthesized a catalyzer based on PdAg@MIL-101 to catalyze the reactants [55]. Pd has great hydrogenation activity, MIL-101 has Lewis acidity, and the target product’s selectivity is significantly increased by Ag (Figure 2). The final catalytic effect is also satisfactory. After introducing different functional groups on the surface of the material, MOFs can still have coordinated unsaturated metal sites, further improving the catalytic activity of MOFs and accelerating the interaction between pollutants and MOFs.

## 3. Synthesis of MOFs Modified by Functional Groups

### 3.1. Direct Synthesis Using Functionalized Linkers

Functionalized MOFs can be synthesized directly by simply using linkers containing various functional groups. For example, we can synthesize materials of the UiO-66(Zr)s series using Zr as the metal center and various types of BDC (benzenedicarboxylate) as the organic ligand [56]. Cu-BTC-NH_2_ (BTC: 1,3,5-benzenetricarboxylate) can be synthesized by using Cu as the metal center and 2-amino-1,3,5-benzenetricarboxylate (BTC-NH_2_) as the organic ligand [57]. The direct synthesis method has the advantages of simplicity and convenience. However, the cost of linkers containing various functional groups leads to the high price of this method. In addition, there are two problems with the use of this method. On the one hand, the synthesis of functionalized MOFs is not easy due to the coordination reaction activity of some functional groups. On the other hand, the reaction products usually have low crystallinity or porosity.

### 3.2. Post-Synthetic Modification

Functionalized MOFs can also be obtained by post-modification methods after the synthesis of the pristine MOFs. The first approach is to take full advantage of the open metal sites on the MOFs. For example, we can use Lewis bases like aminoethanol, aminobutane, ethylenediamine, and others to graft onto the open metal sites of MOFs like MIL-101(Cr), MIL-100(Al, Cr, Fe, V), MOF-74s, and others, thereby introducing -NH_2_ [58,59]. It should be noted that some well-crystallized MOFs do not have open metal sites [60,61,62]. If they are functionalized using a post-modification approach, the effect is not that significant. An alternative approach is to functionalize MOFs through a multi-step process. This involves chemical reactions between pristine MOFs and organic ligands. For example, MOF-NH_2_ can react with butane sulfonic acid, oxalyl chloride, and hydroquinone chloride to introduce -(CH_4_)_2_-SO_3_H, -CO-COOH, and -CO-C_6_H_4_-COOH, respectively [63,64].

### 3.3. Other Means

In addition to the two main categories mentioned above, we can also use other methods to functionalize MOFs. We’ll take MIL-101(Cr) as an example. MIL-101(Cr) can be oxidized to introduce -NO_2_ on the benzene ring, and then MIL-101(Cr)-NO_2_ can be reduced further to MIL-101(Cr)-NH_2_ [65]. In addition, -SH introduced onto MIL-101(Cr) by grafting can be oxidized to -SO_3_H [66]. MIL-101(Cr)-SO_3_H, which is directly synthesized by monosodium 2-sulfoterephthalic acid, could further react with AgNO_3_ to produce MIL-101(Cr)-SO_3_Ag [67,68].

## 4. Mechanism of Contaminant Adsorption in MOFs Modified by Functional Groups

In this part, we mainly introduce the mechanisms of adsorption of pollutants by MOFs after the introduction of functional groups. The interactions are mainly divided into the following five types: Electrostatic interaction, hydrogen-bond interaction, π–π interaction, hydrophobic interaction, and Lewis acid–base interaction.

### 4.1. Electrostatic Interaction

Electrostatic action is one of the most familiar principles for pollutant treatment. Electrostatic force refers to the interaction between charged bodies at rest. This interaction usually occurs via the surfaces of an object. As adsorbents, MOFs and pollutants with opposite charges may attract each other, resulting in electrostatic interaction, so as to achieve the effect of water purification. It is worth noting that zeta potential is an important factor influencing electrostatic action [69]. It depends on the solution pH value. The higher the pH value, the more negative charge will be on the surface. Oppositely, the lower the pH value, the more positive charge will be on the surface. [70]. For example, Wang et al. synthesized UiO-66-NH_2_ for Pb^2+^ and Cd^2+^ adsorption. The higher the pH value, the stronger the adsorption capacity of the material for Pb^2+^ and Cd^2+^ [71]. This can be explained by the point of zero charge (PZC). By studying the diagram of pH and zeta potential, we can see that the value of PZC is 3.86. When pH > PZC, the MOFs are negatively charged, which may increase the electrostatic interaction between the surface of the MOFs and the cations, resulting in increased adsorption of Pb^2+^ and Cd^2+^. On the contrary, when pH < PZC, the adsorption of Pb^2+^ and Cd^2+^ by MOFs with positive charges decreases. Additionally, Ibrahim et al. used UiO-66-NH_2_@SiO_2_ to study its adsorption capacity for Cr_2_O_7_^2−^, methyl orange (MO), and methylene blue (MB) [72]. The relational graph of zeta potential and pH indicates that the PZC of MOF material is 5.6. When the pH was adjusted to 5.4 (Cr_2_O_7_^2−^), 3.5 (MO), and 9.7 (MB), UiO-66-NH_2_@SiO_2_ exhibited ideal adsorption efficiency for the three pollutants. Since both Cr_2_O_7_^2−^ and MO are negatively charged, lowering the pH promotes their adsorption by MOFs. MB is a cationic dye, so increasing the pH value can make MOFs negatively charged so that MB can be adsorbed by electrostatic interaction. Therefore, we need to reasonably regulate the solution’s pH value according to the properties of MOFs by introducing corresponding functional groups so as to achieve effective adsorption of pollutants.

### 4.2. Hydrogen-Bond Interaction

Hydrogen bonding is widely regarded as a vital principle in biochemical or organic communities [73]. The hydrogen-bond is a bond that is weaker than a chemical bond and stronger than intermolecular force. The intermolecular force, also known as van der Waals’ force, is a weak interaction that exists between molecules. It can be mainly divided into dispersion force, orientation force and induction force. (ΔG _hydrogen-bond_: 25–40 KJ·mol^−1^, ΔG _chemical bond_: >100 KJ·mol^−1^, ΔG _van der Waals’ force_: 2–20 KJ·mol^−1^). Hydrogen-bond is caused by the interaction of hydrogen atoms with highly electronegative atoms (O, N, S, F, etc.) [74]. This is usually done when dealing with organic pollutants. Functional groups containing hydrogen atoms modified on MOFs (such as -NH_2_, -OH, etc.) can, for example, hydrogen-bond with some heterocyclic compounds (such as pyridine) [75].

### 4.3. π–π. Interaction

The π–π interaction is related to the interaction between π orbitals in molecules. The π orbital is the molecular orbital produced by the formation of the π bond. This is a weak interaction that usually exists between aromatic rings. It often occurs between two molecules that are electron-deficient and electron-rich and is not a covalent bond interaction as important as hydrogen bonding [76]. Most of the organic ligands used to synthesize MOFs are compounds containing aromatic rings (such as terephthalic acid, phthalic acid, homophenic acid, etc.). They can achieve π–π interaction with pollutants in water containing aromatic rings to form a tight, layered structure that can effectively resist the influence of external factors, allowing the two molecules to stay together.

### 4.4. Hydrophobic Interaction

Hydrophobicity is a physical property of the surface of advanced materials. Hydrophobic materials are made up of molecules with long carbon chains and low water solubility. MOFs are a typical class of hydrophobic materials, most of which tend to be non-polar solvents (such as saturated hydrocarbons and benzene compounds). In the presence of MOFs, non-polar substances aggregate in the water solution and tend to exclude water molecules [77]. Using this effect, MOFs can be successfully applied to pollutant removal. For example, Lin et al. used HKUST-1 to remove oil droplets from solution, and the interpretation of the result showed that the adsorption capacity of MOF material to oil droplets reached 4000 mg·g^−1^ [78]. This is primarily due to the strong hydrophobic action of the benzene ring in the oil droplets and HKUST-1. Yang et al. reported a particularly hydrophobic porous material (FMOF-1) with high adsorption capacities for benzene, cyclohexane, *n*-hexane, *p*-xylene, and toluene. At 100% relative humidity, no adsorption of water was detected [79].

### 4.5. Lewis Acid–Base Interaction

Lewis’ theory of acid and base was first proposed by American chemist Gilbert N. Lewis. This theory defines that any substance that can accept electron pairs is called an acid, and any substance that can give them is called a base [80]. A Lewis acid–base reaction is a reaction in which a coordination covalent bond is formed between an electron-pair acceptor and an electron-pair donor. This theory plays a key function in the MOF-based adsorption course. For instance, we can modify some groups with Lewis basic properties (such as -NH_2_, -SH, etc.) on the original MOF material and capture heavy metal ions from water using the soft and hard acid–base theories [81,82].

## 5. Application of MOFs by Modified Functional Groups in Water Purification

In this part, we classify MOFs according to different functional groups and introduce the MOFs’ specific application in the purification of water after introducing common functional groups in detail. The corresponding adsorption results and adsorption mechanism are arranged in Table 1 for the convenience of readers.

### 5.1. N-Containing Group

#### 5.1.1. -NH_2_

**Figure 3:**
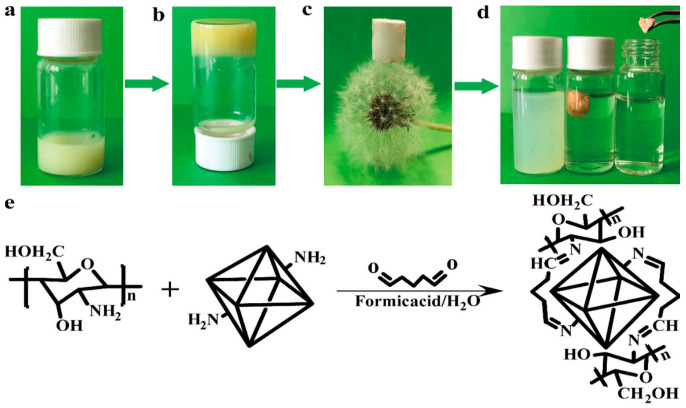
(**a**–**c**) Synthesis route of UiO-66-NH_2_-CS aerogel (**d**) Adsorption of Pb^2+^ by UiO-66-NH_2_-CS (**e**) Synthesis equation of UiO-66-NH_2_-CS. Reprinted with permission from Ref. [83]. Copyright 2019, Elsevier.

Liu et al. synthesized UiO-66-NH_2_-CS aerogel monomers with hierarchical structure by covalent cross-linking, which showed effective and stable adsorption of Pb^2+^ [83]. The aerogel was characterized by many pores and a low density. The adsorption of Pb^2+^ by the material was 102.03 mg·g^−1^. Among them, N and Pb^2+^ play a coordination role, and O plays a synergistic adsorption role to a certain extent. After three cycles, the material’s adsorption capacity remained at 90.12%. This strategy provides an effective and universal approach for using MOFs in the field of pollutant treatment (Figure 3).

**Figure 4:**
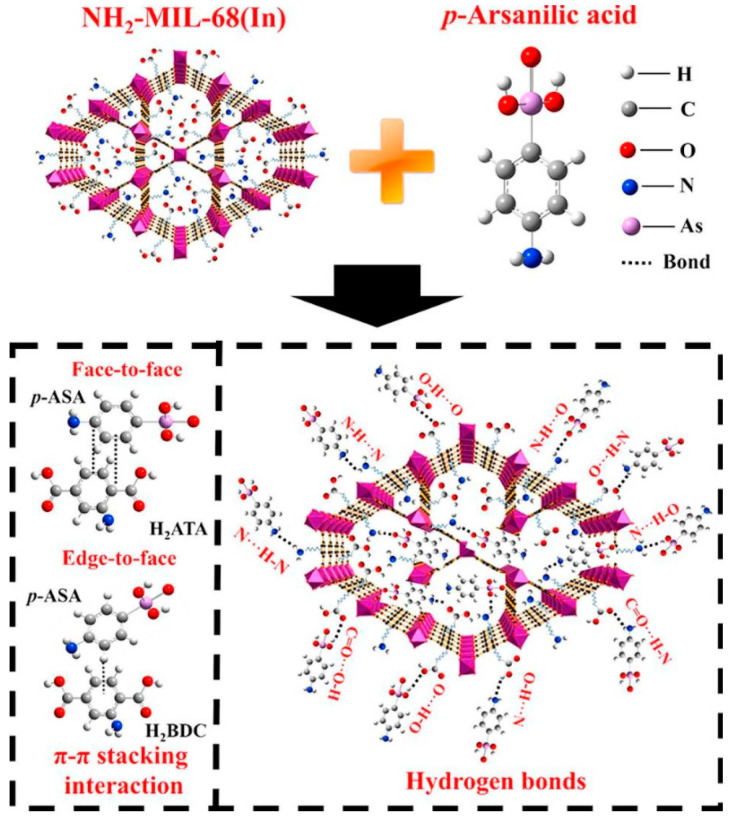
Structural formula of NH_2_-MIL-68(In) and main adsorption mechanism of *p*-ASA by NH_2_-MIL-68(In). Reprinted with permission from Ref. [89]. Copyright 2018, Elsevier.

Azmi et al. proposed a new synthesis method to prepare MIL-96, which has different crystal habits and a larger particle size [84]. After synthesizing the material, they added hydrolyzed polyacrylamide (HPAM) to increase the size of the material. Activated carbon (AC) was used as a reference adsorbent to examine the removal impact of MIL-96-RHPAM2 on perfluorooctanoic acid (PFOA). The results showed that MIL-96-RHPAM2 had a good PFOA adsorption capability of 340 mg·g^−1^. The amine group and the anionic carboxylate of PFOA in the HPAM skeleton generate electrostatic bonds, which contribute to the high adsorption of PFOA by MIL-96-RHPAM2. Samuel et al. synthesized Cu-BTC@NH_2_ composites and investigated their adsorption capacity for two drug contaminants, ibuprofen (IBF) and acetaminophen (ACE), in aqueous solutions [85]. The course of adsorption accords with the pseudo-second-order kinetic model and Langmuir adsorption, which reveal that chemisorption is the prime adsorption process. Based on hydrophobic and electrostatic effects, the adsorption capacities of IBF and ACE were 187.97 mg·g^−1^ and 125.45 mg·g^−1^, accordingly. The laboratory findings display that the approach can be applied to eliminate harmful drugs from toxic wastewater. In a study, Park et al. used MIL-101 to adsorb bisphenol S (BPS) in water and investigated the effectiveness of the introduction of -NH_2_ on adsorption [86]. The elimination capacity of BPS was greatly enhanced, and the adsorption capacity reached 513 mg·g^−1^ when the -NH_2_ group was added to MIL-101. The research reveals that hydrogen bonds exist in MIL-101-NH_2_, as well as O=S=O and -OH in BPS. The material can still have good adsorption characteristics after routine ethanol washing. The method also provides a novel horizon for the adsorption of organics containing sulfonyl in wastewater. Wu et al. compounded an adsorbent (UiO-66-NH_2_) that can efficiently remove Cr^6+^ from water [87]. According to the pseudo-second-order kinetic model, the initial stage of Cr^6+^ adsorption is rapid, and the subsequent adsorption phase is slow. The adsorption capacity of Cr^6+^ was determined by the Langmuir model to be 32.36 mg·g^−1^, and it occurred spontaneously at pH = 6.5. The electrostatic attraction between Cr^6+^ and UiO-66-NH_2_ may be the principle of Cr^6+^ adsorption by UiO-66-NH_2_. Roushani et al. demonstrated in an experiment that TMU-16-NH_2_ is a resultful adsorbent for the treatment of Cd^2+^ from water solutions [88]. The results show that the initial concentration and pH of the solution have a great influence on the adsorption, which is spontaneous. The adsorbent’s maximal Cd^2+^ adsorption capacity was 126.6 mg·g^−1^. Mechanism studies show that the amino group of TMU-16-NH_2_ is a good binding site for Cd^2+^, and the adsorption of Cd^2+^ is facilitated by the creation of coordination bonds between -NH_2_ groups and Cd^2+^. The performance of the adsorbent has been greatly improved compared with the results of research. Lv et al. used two MOFs (original MIL-68(In) and NH2-MIL-68(In)) to investigate the adsorption efficiency of typical organic arsenic compounds, arsine acid (*p*-ASA). [89]. The adsorption quantity of NH_2_-MIL-68(In) for *p*-ASA (401.6 mg·g^−1^) is greater than that of pure MIL-68(In), which can be attributed to hydrogen bonding and the π–π effect (Figure 4). In addition, the result of the adsorption of *p*-ASA is exothermic and spontaneous, conforming to the pseudo-second-order intraparticle diffusion model. The addition of -NH_2_ to MIL-68(In) accelerates adsorption performance by increasing hydrogen bonding. This research offers a crucial theoretical and experimental foundation for the use of MOFs in the elimination of organic arsenic compounds like *p*-ASA.

**Figure 5:**
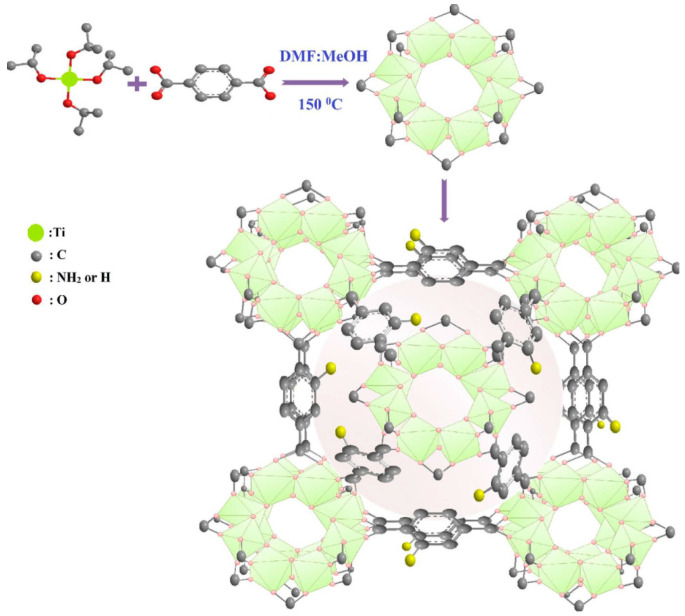
Synthesis and structure diagram of NH_2_-MIL-125(Ti). Reprinted with permission from Ref. [93]. Copyright 2017, Elsevier.

Roushani et al. investigated the adsorption and removal of the anionic dye methyl orange (MO) by two MOF materials (TMU-16-NH_2_ and TMU-16) [90]. Thermodynamic and kinetic outcomes display that the removal of MO by the two MOFs is a spontaneous pseudo-second-order adsorption, and the removal rate of MO by TMU-16-NH_2_ (393.7 mg·g^−1^) is superior to that of the original TMU-16 because the hydrogen-bond between MO and -NH_2_ is formed and the material itself is stable. The adsorption properties of these materials for anionic dyestuffs are stronger than those of other previously reported porous materials. Electrostatic interaction and hydrogen bonding both contribute to the understanding of the adsorption mechanism. Haque et al. prepared two metal-organic frameworks, NH_2_-MIL-101(Al) and MIL-101(Al) [91]. These two materials have been applied to dislodge methylene blue (MB) from aqueous solutions. The adsorption capacity of NH_2_-MIL-101(Al) on MB at 30 °C was up to 762 mg·g^−1^ due to the electrostatic interaction between the cationic dye MB and the amino group of MOFs. The original MIL-101(Al) exhibited lower adsorption capability, which explains why -NH_2_ was introduced. Liu et al. put forward a magnetic MOF substance for the adsorption of several cationic and anionic dye types [92]. Adsorption isotherm and thermodynamic studies showed that the maximum adsorption capabilities of NH_2_-MIL-101(Al) for indigo carmine (IC) and malachite green (MG) were 135 mg·g^−1^ and 274.4 mg·g^−1^, respectively. This is primarily the contribution of π–π interaction, electrostatic interaction, hydrogen bonding and hydrophobic interaction between magnetic NH_2_-MIL-101(Al) and organic dyes. The method has the characteristics of a short separation time and a high reuse rate, which is expected to make it a new adsorbent for adsorbing and removing dyes from aqueous solutions. Oveisi et al. used Ti as a metal source, 2-amino-1,4-phthalate ester (NH_2_-BDC), and 1,4-phthalate ester (BDC) as organic connectors to synthesize five kinds of materials in the MIL series, which are MIL-X1, MIL-X2, MIL-X3, NH_2_-MIL-125(Ti), and MIl-125(Ti) (the first three materials are obtained by adjusting the ratio of BDC to NH_2_-BDC, respectively) [93] (Figure 5). These materials were used to study the removal of the cationic dyes methylene blue (MB), basic blue 41 (BB41), and basic red 46 (BR46) from water. The adsorption of pollutants by the five materials of the MIL series conforms to the pseudo-second-order kinetic model and the Langmuir adsorption, and the synthesized MIL has high reuse and stability within the three cycle periods. Most importantly, the maximum electron density was found in NH_2_-MIL-125(Ti), so the adsorption performance is particularly ideal. The adsorption of MB, BB41, and BR46 is respectively 862, 1257, and 1296 mg·g^−1^.

#### 5.1.2. -NMe^3+^

Wu et al. created a novel quaternary amine anion-exchange MOF (UiO-66-NMe^3+^) for the adsorption of 2,4-dichlorophenoxyacetic acid (2,4-d), a toxic pesticide that is extensively utilized [94]. UiO-66-NMe^3+^ had a maximum adsorption capacity for 2,4-d of 279 mg·g^−1^, which was much higher than UiO-66 and UiO-66-NH_2_. This is mainly attributed to the following two reasons. For one thing, the functionality of quaternary amine groups can effectively increase the electrostatic effect. For another, the π–π conjugation between 2,4-d and MOFs improves the adsorption properties of UiO-66-NMe^3+^. After seven cycles, the adsorption performance decreased only slightly. This technique offers the best alternative for effectively removing 2,4-d from complex water conditions by adsorption. Wei et al. created the functionalized quaternary ammonium salt MIL-101(Cr)-NMe^3+^ and took advantage of it as a novel adsorbent to wipe off diclofenac sodium (DCF) from wastewater [95]. The results showed that DCF quickly adsorbed onto MIL-101(Cr)-NMe^3+^. At 20 °C for 30 min, DCF can be adsorbed in large quantities (310.6 mg·g^−1^), which conforms to the pseudo-second-order kinetic model and the Langmuir adsorption. According to the adsorption mechanism analysis, the electrostatic interaction and π–π interaction between MIL-101(Cr)-NMe^3+^ and DCF are the reasons for the efficacious adsorption. Based on the results of the regeneration experiment, MIL-101(Cr)-NMe^3+^ could be recovered at least five times without suffering a substantial decrease in adsorption ability.

Based on the preceding discussion, we can conclude that MOFs modified with -NH_2_ or -NMe^3+^ can be useful in water purification. For organic matter in water (such as organic poisons, dyes, and drugs), the general adsorption mechanism focuses on hydrogen bonding and electrostatic interaction. This is very understandable. Because N is a highly electronegative atom, it can be used as the acceptor of H, thus forming hydrogen bonds and realizing the adsorption of pollutants. For heavy metal ions in water, -NH_2_ successfully acts as a Lewis base. Because metal ions are Lewis acids, they can interact with -NH_2_ in Lewis acid–base reactions to produce the adsorption effect.

### 5.2. S-Containing Group

#### 5.2.1. -SH

**Figure 6:**
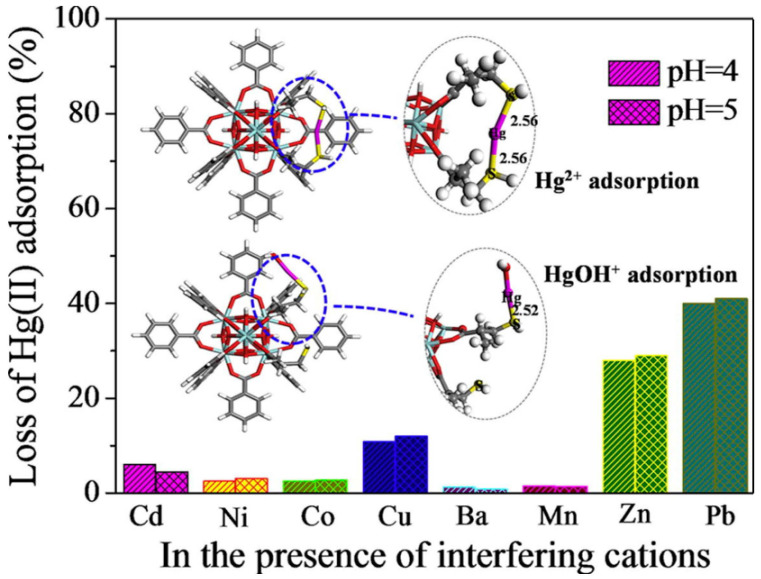
Schematic diagram of UiO-66-SH selective adsorption of Hg^2+^ at different pH values. Reprinted with permission from Ref. [97]. Copyright 2018, Elsevier.

Ke et al. selected the classic 3D Cu-MOF (HKUST-1) and used a simple post-synthesis strategy to achieve sulfhydryl functionalization of MOFs to investigate their application in removing Hg^2+^ from water [96]. They prepared a sequence of sulfhydryl-modified HKUST-1 by ligand-bonding the coordination unsaturated metal center in HKUST-1 to the -SH group in disulfide glycol. Under the same conditions, sulfhydryl-functionalized HKUST-1 showed very high adsorption capacity (714.29 mg·g^−1^) and fine adsorption affinity for Hg^2+^ in water. Nevertheless, unfunctionalized HKUST-1 demonstrated no adsorption for Hg^2+^. This method is considered to be a new and effective method for the treatment of heavy metal ions in water. Li et al. created sulfhydryl-modified MOFs (UiO-66-SH) by a simple tactic and used them for selectively extracting Hg^2+^ in aqueous solutions [97]. After the introduction of -SH, the rate of UiO-66-SH’s adsorption to Hg^2+^ was extremely rapid, showing high adsorption performance (3.91 mmol·g^−1^) in a broad pH range (2.3–8.0), and the adsorption efficiency remained high (> 90%) after seven regenerations. Even though there are other heavy metal ions (Cu^2+^, Mn^2+^, Ni^2+^, Cd^2+^, Ba^2+^, and Co^2+^), the adsorbent exhibited selective adsorption of Hg^2+^ (Figure 6). Density functional theory (DFT) calculations revealed that this was primarily due to the strong coordination between Hg^2+^ and -SH. It is worth mentioning that UiO-66-SH possesses a unique attraction for organic mercury forms as well. (PhHg^+^, EtHg^+^, and MeHg^+^). This method can provide a new idea for removing mercury from ecological environments.

**Figure 7:**
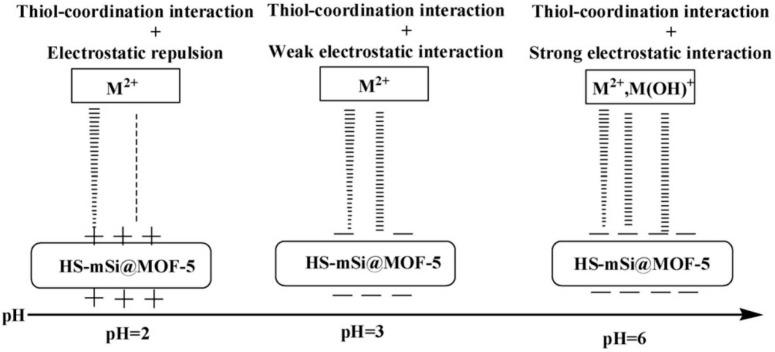
Schematic diagram of the strength of HS-mSi@MOF-5 on metal ions at different pH values. Reprinted with permission from Ref. [99]. Copyright 2016, Elsevier.

By functionalizing MIL-88A with mercaptoethanol, Singh et al. created MIL-88A-SH, a new-style MOF-based adsorbent, and tested its removal of Hg^2+^ from water and Hg from air [98]. The absorption is constant in the pH range of 5.0–9.0, and the adsorption capacity of Hg^2+^ is very high, about 1111.1 mg·g^−1^. In addition, when there are other interfering metal ions (Cu^2+^, As^3+^, Zn^2+^, Cr^6+^, Pb^2+^, and Cd^2+^), MIL-88A-SH still shows good adsorption properties for Hg^2+^. For Hg in the air, about 45.6 mg·g^−1^ of Hg^2+^ is adsorbed. This is mainly because Hg was oxidized to Hg^2+^ and complexed with sulfhydryl groups during the adsorption process. These data indicate that MIL-88A-SH can be used as a highly effective adsorbent to solve mercury pollution. Zhang et al. made use of the hydrothermal method to create MOF-5. On this basis, mercaptan-functionalized MOFs (HS-mSi@MOF-5) were invented and applied to research the adsorption effects of Pb^2+^ and Cd^2+^ in water [99]. In view of the capacity and rate of adsorption, the adsorption performance of HS-mSi@MOF-5 (312.5 mg·g^−1^, 65.2 mg·g^−1^) is preferable to that of the original material (211 mg·g^−1^, 4.2 mg·g^−1^). In the water stability test, the unmodified MOF-5 will gradually dissolve in an acidic solution, but its stability increases after the introduction of -HS. Based on the mechanism of adsorption, the coordination interaction and electrostatic interaction between -HS and toxic metal ions are critical for the disposal of Cd^2+^ and Pb^2+^ (Figure 7).

#### 5.2.2. -SO_3_H

**Figure 8:**
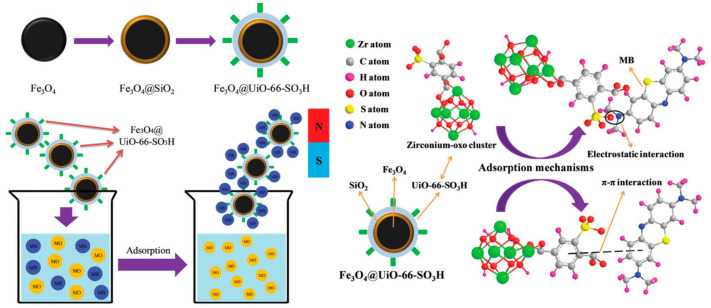
Schematic diagram of selective adsorption of MB by Fe_3_O_4_@UiO-66-SO_3_H and corresponding adsorption mechanism. Reprinted with permission from Ref. [104]. Copyright 2019, Royal Society of Chemistry.

Moradi et al. synthesized and used sulfonated MOFs supported on Fe_3_O_4_ NPs as a Fenton-like catalyst to remove methyl orange (MO) from water [100]. When the initial concentration is 100 mg·L^−1^, the incipient concentration of H_2_O_2_ is 40 mg·L^−1^, the microwave power is 500 W, and the pH value is 3.0, the rate of the elimination of MO is very fast. Only when the microwave radiation time is 6 min, as much as 99.9% of MO can be removed. Finally, microwave-induced Fe_3_O_4_@MIL-100(Fe)-SO_3_H degradation will be a wastewater dye removal technique with great promise. Hasan et al. first synthesized three Zr-MOF series materials (UiO-66-SO_3_H, UiO-66-NH_2_, and UiO-66) for the removal and adsorption of diclofenac sodium (DCF) in water and contrasted these three materials with activated carbon (AC) [101]. These three materials showed better adsorption performance than AC. The adsorption kinetics between UiO-66 and DCF are faster due to possible π–π interaction and electrostatic interaction. After the introduction of -SO_3_H, the kinetics and capacity (263 mg·g^−1^) of adsorption were significantly enhanced, which may result from the acid–base attraction of -SO_3_H in UiO-66-SO_3_H and -NH_2_ in DCF. Interestingly, however, the opposite trend was observed when UiO-66-NH_2_ was utilized as a sorbent. This is primarily due to the alkali-base rejection of -NH_2_ in DCF and UiO-66-NH_2_, on account of relevant results. Wang et al. immobilized -SO_3_H on the surface of MOFs to synthesize sulfonic acid-functionalized MOFs to remove Cd^2+^ from aqueous solutions [102]. Cu_3_(BTC)_2_-SO_3_H showed a relatively good absorption capacity of 88.7 mg·g^−1^, exceeding that of the reference adsorbent. Additionally, it has quick kinetics for the adsorption of Cd^2+^ from aqueous solutions that are 1–3 orders of magnitude higher than those of current adsorbent materials. Furthermore, despite the existence of other interfering ions, it indicates a strong selectivity for Cd^2+^ and is simple to regenerate and recycle without suffering a major reduction in Cd^2+^ adsorption capacity. Liu et al. focused on the adsorption behavior of UiO-66@mSi-SO_3_H, UiO-66@mSi-SH, and UiO-66 on Cd^2+^ [103]. In consideration of the data of the Elovich and the Langmuir models, the adsorption capacity can be UiO-66 < UiO-66@mSi-SH < UiO-66@mSi-SO_3_H. The theoretical maximum adsorption capacities of UiO-66@mSi-SO_3_H and UiO-66@mSi-SH for Cd^2+^ are 409.96 and 212 mg·g^−1^, respectively. In the presence of -SH and -SO_3_H, Cd^2+^ in water was replaced by adsorbents to accomplish the aim of getting rid of Cd^2+^. -SO_3_H has diverse and complex coordination forms, allowing UiO-66-mSi-SO_3_H to have an excellent adsorption capacity for removing Cd^2+^. Lv et al. synthesized a late-model magnetic nanocomposite with a core-shell structure of Fe_3_O_4_@UiO-66-SO_3_H by means of a viable stepwise assembly tactic and then made the most of it as an adsorbent to wipe off methylene blue (MB) from aqueous solutions [104]. A considerable affinity for MB may be shown in the synthesized Fe_3_O_4_@UiO-66-SO_3_H, which has a maximum adsorption capacity of 297.3 mg·g^−1^. The adsorption process is basically completed within 15 min. Because of the effect of charge selectivity, Fe_3_O_4_@UiO-66-SO_3_H has good reusability and can selectively adsorb MB from the mixed solution. The π–π interaction and electrostatic interaction are important during the adsorption process. (Figure 8).

#### 5.2.3. -SO_4_

**Figure 9:**
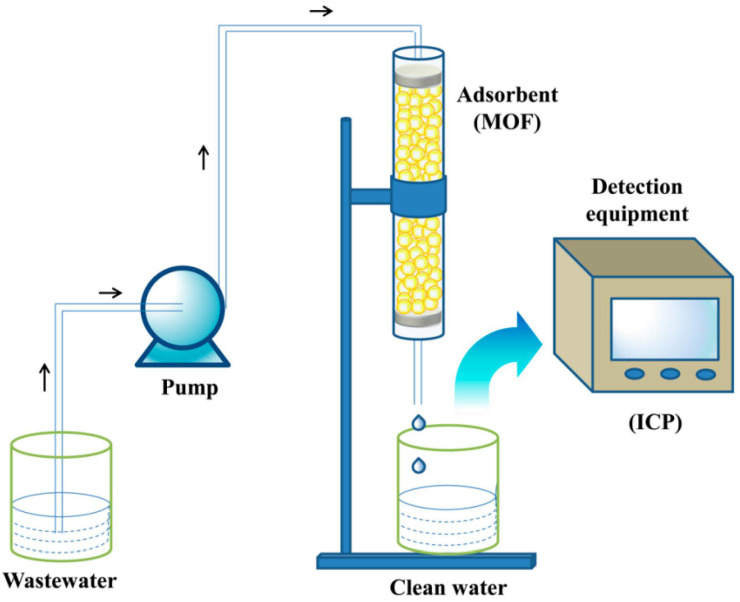
Schematic diagram of experimental setup used for breakthrough experiments. Reprinted with permission from Ref. [105]. Copyright 2017, American Chemical Society.

Kang et al. selected Zr-BTC-NH_2_-SO_4_ as an adsorbent to capture radioactive Ba^2+^ in nuclear wastewater [105] (Figure 9). By reason of the plentiful -SO_4_ groups in the skeleton, which are strong Ba^2+^ chelating groups, and the fact that the binding site is completely exposed, in comparison to most adsorbents, Zr-BTC-NH_2_-SO_4_ has a greater adsorption capacity of 181.8 mg·g^−1^. When the concentration of interfering ions is ten times that of Ba^2+^, the material still shows excellent selectivity. Most crucially, the course of adsorption is irreversible for Ba^2+^, which can availably avert secondary pollution. This work has contributed to the development of adsorbents for the handling of radioactive Ba^2+^ from nuclear effluent.

Peng et al. proposed a Ba^2+^ defect concept based on MOFs by introducing -SO_4_ functional groups into the pore structure of MOFs [106]. The functionalized MOFs can remove more than 90% of Ba^2+^ within 5 min, and the removal efficiency reaches 99% after equilibrium. Notably, the sulfate-based functionalized material showed a high Ba^2+^ absorption capacity of 131.1 mg·g^−1^, exceeding that of most reportorial adsorbents, and could selectively capture Ba^2+^ from outlet water. This paper offers a fresh viewpoint on environmental remediation and the removal of radioactive Ba^2+^ from nuclear effluent.

MOFs modified with functional groups containing S atoms are generally used in the catalytic field, but due to some special properties, they can also be used to remove pollutants from water sources [117]. For S-containing groups, -SH, -SO_3_H, and -SO_4_ are the most representative. Since these three are nucleophiles, they can be regarded as Lewis bases, which explains their ability to adsorb metal ions from water through the Lewis acid–base process. For -SO_3_H, when it is modified into organic molecules, it can increase the acidity of an aqueous solution, making the pH drop, thus promoting the electrostatic interaction between it and pollutants. In addition, -SO_3_H is a hydrophilic group, which can increase the water solubility of MOFs. For -SH, it can also produce the same effect as -SO_3_H, but the acidity is not that strong, so the electrostatic effect of -SH is weaker than that of -SO_3_H. In addition, thanks to the presence of S atoms, MOFs modified by functional groups can also form hydrogen bonds with organic matter, but the effect is less obvious than the previous two.

### 5.3. -OH and -COOH

#### 5.3.1. -OH

**Figure 10:**
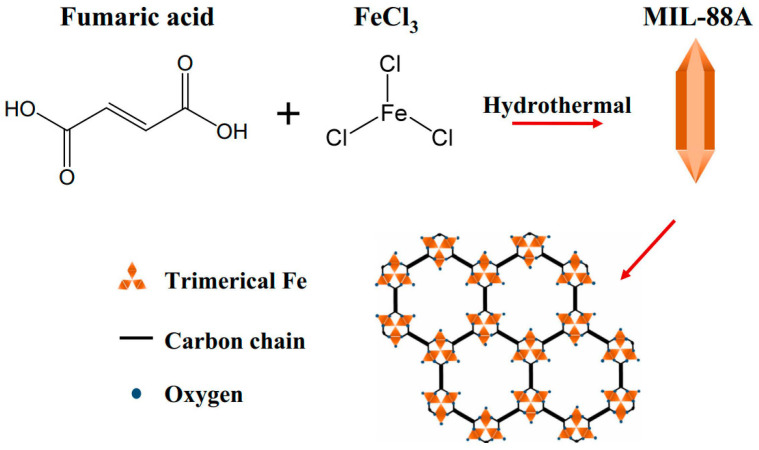
Synthesis method and structure diagram of MIL-88A nanorods. Reprinted with permission from Ref. [107]. Copyright 2018, Springer Nature.

Wu et al. synthesized MIL-88A nanorods by the hydrothermal method, which was first used to deal with As^5+^ in water [107]. The chemical process conforms to the Langmuir adsorption model and the pseudo-second-order kinetic model, indicating that the adsorption of MIL-88A on As^5+^ is chemical adsorption and monolayer adsorption. In consideration of its distinctive structure, which contains several -OH groups and has a high swelling to water, MIL-88A has a maximum adsorption capacity of 145 mg·g^−1^. These results indicate that MIL-88A can be regarded as a fantastic candidate for arsenic removal (Figure 10).

Seo et al. used MIL-101-(OH)_2_, MIL-101-OH, MIL-101-NH_2_, and MIL-101 to study the adsorption of ibuprofen, naproxen, and oxybenone in water [108]. Among them, naproxen adsorption is the most representative. The fact that MIL-101 with H donor functional groups like -OH and -NH_2_ is particularly effective in adsorbing naproxen may be because the O atom on the drug and the H atom in the adsorbent have formed hydrogen bonds. The more hydrogen bonds contained, the more significant the adsorption effect, which also explained why MIL-101-(OH)_2_ had the best adsorption effect on naproxen (185 mg·g^−1^). In addition, MIL-101-(OH)_2_ can be recovered multiple times by simply washing it with ethyl alcohol, suggesting its potential for adsorption-based removal of PPCPs in water. Song et al. explored the effectiveness of hydroxyl groups on the adsorption of 5 PPCPs (bisphenol A, *p*-chloro-m-xylenol, naproxen, ketoprofen, and triclosan) on MIL-101 [109]. The hydrogen-bond is considered to be the main mechanism of adsorption of PPCPs on MOFs, in which MOFs and PPCPs can be H donors and H acceptors, respectively. This result is based on the fact that the greater the number of H receptors in PPCPs and the greater the number of -OH groups in MOFs, then the higher the adsorption capacity. This phenomenon is particularly significant for the adsorption of MIL-101-(OH)_3_ to chloroxylenol, ketoprofen, and naproxen. Sun et al. adopted a hydrothermal approach to create a novel adsorbent UiO-66-(OH)_2_/GO nanomaterial [110]. The excellent adsorption feature of UiO-66-(OH)_2_/GO on tetracycline hydrochloride (TC) and methylene blue (MB) pollutants (37.96 mg·g^−1^, 96.69 mg·g^−1^) confirmed the excellent adsorption performance of UiO-66-(OH)_2_/Go. The Freundlich isothermal model and the pseudo-second-order kinetic model demonstrate that the adsorption is chemisorption and single-layer adsorption, and these adsorption processes are completely spontaneous. These findings imply that UiO-66-(OH)_2_/GO offers valuable information on the production of sorbent materials for purifying water.

#### 5.3.2. -COOH

**Figure 11:**
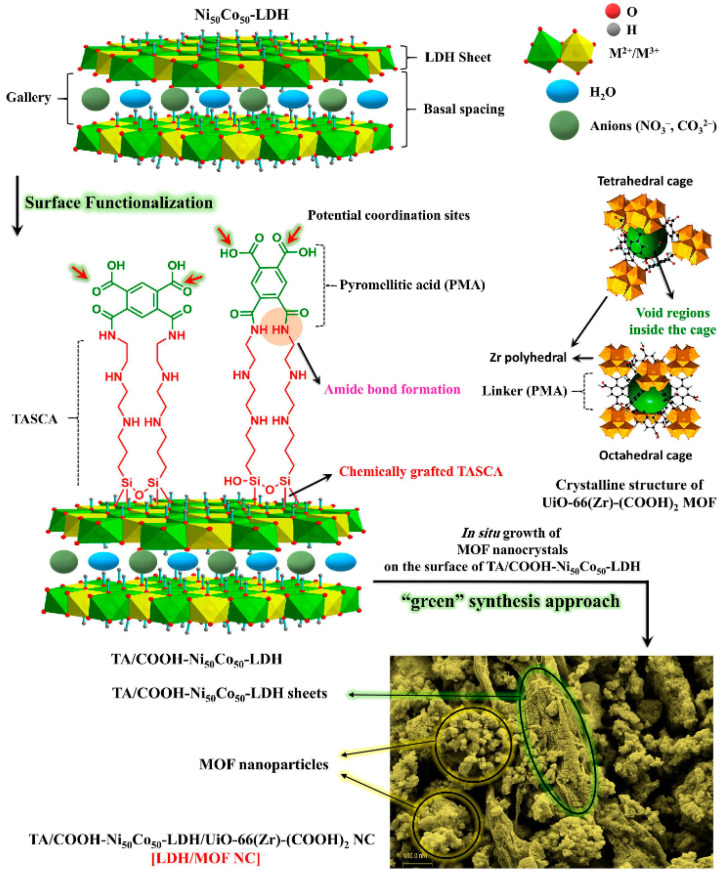
The overall synthesis process of the LDH/MOF NC. Reprinted with permission from Ref. [113]. Copyright 2021, Elsevier.

Zhu et al. produced a recyclable MOF-based nanoparticle, UiO-66-2COOH, and created biochar from readily available waste biomass to remediate Sb^3+^ contamination in water by combining the two [111]. The adsorption capacity was 56.49 mg·g^−1^ when pH = 9.1, at 70 °C for 4 h, the initial concentration was 100 mg·L^−1^, and the mass ratio of UiO-66-2COOH to biochar was 4:1. The Freundlich isotherm model and the pseudo-second-order kinetic model were also studied. In the presence of NO_3_^−^, SO_4_^2−^, Cl^−^, and PO_4_^3−^, the adsorbent has no influence on the adsorption of Sb^3+^. Ren et al. reported the stable double-group MOF (Zr-BTC-COOH-SO_4_), a skeleton containing strontium chelating groups (-SO_4_, -COOH) and consumingly ionized groups (-COOH) [112]. The greatest sorption was 67.5 mg·g^−1^ for less than 5 min. It is worth mentioning that solution pH and adsorption temperature have no obvious effect on the adsorption of Sr^2+^ by Zr-BTC-COOH-SO_4_. The adsorption mechanism is that -COOH results in prompt adsorption based on electrostatic interaction, and the introduction of -SO_4_ improves the sorption capacity to a great extent. These results indicate that Zr-BTC-COOH-SO_4_ is a probable candidate for Sr^2+^ removal. Soltani et al. prepared the functionalized nanocomposite LDH/UiO-66-(Zr)-(COOH)_2_ by a simple “green” synthesis scheme and employed it as a powerful adsorbent to dislodge Hg^2+^ and Ni^2+^ from aqueous media [113] (Figure 11). It was found that the optimum Langmuir sorption capacity was 509.8 mg·g^−1^ (Hg^2+^) and 439 mg·g^−1^ (Ni^2+^) under constant conditions. The existence of -COOH makes the surface of the material have ample active sites, which improves the adsorption performance to a certain extent. This strategy has contributed to the design of more environmentally friendly and safer heavy metal ion adsorbents.

From the above discussion, we know that -OH and -COOH are both Lewis bases, which can be combined with metal ions by Lewis acid–base action to form coordination covalent bonds. In addition, both -OH and -COOH contain O, which is a highly electronegative element and thus can form hydrogen bonds with many antibiotics (many of which contain O, N, F, Cl, etc.). Since the bond energy of the hydrogen-bond can be regarded as electrostatic interaction to some extent, electrostatic interaction is also the adsorption mechanism of pollutants adsorbed by -OH and -COOH in water systems. The presence of -OH and -COOH increased the active sites of chemical reactions and further increased the adsorption capacity.

### 5.4. Halogen Groups

**Figure 12:**
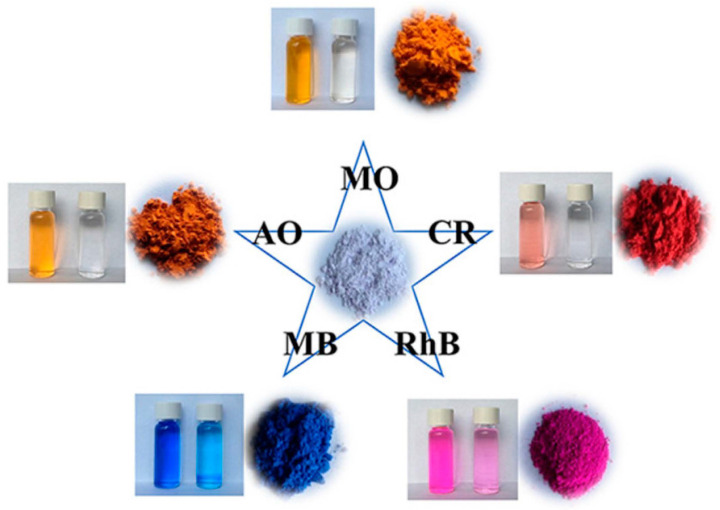
Schematic diagram of adsorption effect of SCNU-Z1-Cl on five organic dyes. Reprinted with permission from Ref. [116]. Copyright 2019, American Chemical Society.

Sini et al. prepared two Zr-MOF-based materials (UiO-66-(F4) and UiO-66) for selective adsorption of PFOA and PFOS from aqueous medium [114]. By characterizing MOFs, they determined how efficiently they absorbed PFOX. In comparison with UiO-66 without modifying the functional groups, UiO-66-(F4) has stronger F-F interaction in the cavity, which increases its adsorption affinity for fluorinated pollutants. Under certain conditions, the saturated adsorption capacity is approximately 470 mg·g^−1^. These findings imply that fluorinated organic contaminants can be pretty much eliminated from aqueous solutions by using Zr-MOFs as a platform. Amador et al. synthesized UiO-66-F4 and UiO-66 using Zr as the metal center and TFBDC (2, 3, 5, 6-tetrafluoro-1, 4-phenyl dicarboxylic acid) and BDC (1, 4-phenyl dicarboxylic acid) as organic joints [115]. These two materials showed excellent adsorption capacity for organic pollutants in water. The hydrophobic material can be obtained by adding -F to the ligand structure of MOFs, which changes the interaction between adsorbate and adsorbent from π–π interaction in the original UiO-66 to hydrophobic interaction in UiO-66-F4. For benzene, UiO-66-F4 (76.3 mg·g^−1^) displayed a more superior adsorption feature than UiO-66 (34.1 mg·g^−1^). Deng et al. synthesized a tubular adsorbent for capturing harmful pollutants in outlet water [116]. SCNU-Z1-Cl has a large specific surface area and high hydrolytic stability in the pH range of 4–10. Because SCNU-Z1-Cl has a large tubular channel and unaligned anions in the skeleton, it can adsorb some anions and anionic dyes by ion exchange. SCNU-Z1-Cl had adsorption capacities of 292, 318, 126, and 241 mg·g^−1^ on MnO_4_^−^, ReO_4_^−^, CrO_4_^2−^, and Cr_2_O_7_^2−^, respectively, which were the highest in the field of MOF/COF. The selectivity of the material remains high even in the presence of other anion species. For organic dyes, the adsorption capacities of Congo red, methyl blue, methyl orange, and acid orange A with different sizes were 585, 262, 285, and 180 mg·g^−1^, within 1 h (Figure 12). SCNU-Z1-Cl can separate organic coloring matter in three disparate patterns: kinetics-dependent selective adsorption, charge-dependent, and size-dependent. The above data fully demonstrate that SCNU-Z1-Cl is an ideal adsorbent that can make outstanding contributions to environmental materials.

At present, the modification of halogen groups on MOFs is rarely studied. Halogens have a strong electronegativity, which can produce an electrical induction effect and increase the hydrophobicity of molecules so as to obtain hydrophobic materials. When the material comes into contact with the solution, it can interact with the contaminants in the water and separate them.

## 6. Summary and Expectation

In summary, MOFs are very effective at adsorbing pollutants from water. On the one hand, the original MOFs themselves possess the features of a large specific surface area, a large and adjustable pore size, and well-coordinated unsaturated metal sites. For another, after modifying MOFs with functional groups (-NH_2_, -NMe^3+^, -SH, -SO_3_H, -SO_4_, -OH, -COOH, -F, -Cl), they give full play to the function of functional groups on the basis of retaining the original characteristics. The above two points further enhance the adsorption performance of MOFs on heavy metal ions, organic dyestuffs, organic poisons, drugs, and acid ions. The main adsorption mechanisms, which represent a critical effect in the specific adsorption process, are electrostatic interaction, π–π interaction, hydrogen bonding, Lewis acid–base interaction, and hydrophobic interaction.

Although there are satisfactory results in pollutant disposal by functional MOFs, we still have several expectations. It is hoped that these opinions will encourage the use of MOF materials in the water purification industry. Firstly, MOFs modified with functional groups require specific binding between specific groups and target objects, which requires priority to be given to group functionalization of reactants. However, there are many conditions for functionalization, and the original framework structure is easily destroyed after functionalization. The adsorption capacity of MOFs’ material on pollutants decreased or even disappeared. Therefore, we should find corresponding solutions. Secondly, the metal centers of MOFs (Cr, Co, Cu, etc.), the organic ligands (imidazoles, carboxylic acids, etc.), and the reaction solvents (DMF, DMAC, etc.) are toxic chemical reagents regardless of the introduction of functional groups. These chemicals are not only expensive but also prone to returning to the environment and causing secondary pollution. Therefore, it is necessary to find green synthesis methods to improve the problem. Thirdly, most of the MOF materials with functional groups need to undergo high temperature and high pressure reactions in the reactor, and the synthesis time is long. We should try to find a method that can synthesize MOFs under mild conditions. Fourthly, at present, the research is in the preliminary stage, and there are not many MOFs applied in water purification. Relevant researchers should continue to explore new MOF materials. Finally, treatment of pollutants with low concentrations is still difficult.

## Figures and Tables

**Table 1:** Application of MOFs modified by functional groups in water purification.

MOF	Introduced Group	Contaminant	Adsorbing Capacity(mg·g^−1^)	Main Adsorption Mechanism	Ref.
UiO-66	-NH_2_	Pb^2+^	102.03	Lewis acid–base interaction	[83]
MIL-96	-NH_2_	PFOA	340.00	Electrostatic interaction	[84]
Cu-BTC	-NH_2_	IBF ACE	187.97 125.45	Electrostatic interaction, Hydrophobic interaction	[85]
MIL-101	-NH_2_	BPS	513.00	Hydrogen-bond interaction	[86]
UiO-66	-NH_2_	Cr^6+^	32.36	Electrostatic interaction	[87]
TMU-16	-NH_2_	Cd^2+^	126.60	Lewis acid–base interaction	[88]
MIL-68(In)	-NH_2_	*p*-ASA	401.60	π–π interaction, Hydrogen-bond interaction	[89]
TMU-16	-NH_2_	MO	393.70	Electrostatic interaction, Hydrogen-bond interaction	[90]
MIL-101(Al)	-NH_2_	MB	762.00	Electrostatic interaction	[91]
MIL-101(Al)	-NH_2_	MG IC	274.40 135.00	Electrostatic interaction, Hydrogen-bond interaction, π–π interaction, Hydrophobic interaction	[92]
MIL-125(Ti)	-NH_2_	BR46 BB41 MB	1296.00 1257.00 862.00	Hydrogen-bond interaction	[93]
UiO-66	-NMe^3+^	2,4-d	279.00	Electrostatic interaction, π–π interaction	[94]
MIL-101(Cr)	-NMe^3+^	DCF	310.60	Electrostatic interaction, π–π interaction	[95]
Cu-MOF	-SH	Hg^2+^	714.29	Lewis acid–base interaction	[96]
UiO-66	-SH	Hg^2+^	784.30	Lewis acid–base interaction	[97]
MIL-88A	-SH	Hg^2+^	1111.10	Lewis acid–base interaction	[98]
MOF-5	-SH	Pb^2+^ Cd^2+^	312.50 65.20	Electrostatic interaction, Lewis acid–base interaction	[99]
MIL-100(Fe)	-SO_3_H	MO	99.9%	Hydrogen-bond interaction, π–π interaction	[100]
UiO-66	-SO_3_H	DCF	263.00	Electrostatic interaction, π–π interaction	[101]
Cu-BTC	-SO_3_H	Cd^2+^	88.70	Lewis acid–base interaction, Electrostatic interaction	[102]
UiO-66	-SO_3_H	Cd^2+^	409.96	Lewis acid–base interaction, Electrostatic interaction	[103]
UiO-66	-SO_3_H	MB	297.30	Lewis acid–base interaction, π–π interaction	[104]
Zr-BTC	-SO_4_	Ba^2+^	181.80	Lewis acid–base interaction	[105]
MOF-808	-SO_4_	Ba^2+^	131.10	Lewis acid–base interaction	[106]
MIL-88A	-OH	As^5+^	145.00	Lewis acid–base interaction	[107]
MIL-101	-OH	NAP	185.00	Hydrogen-bond interaction	[108]
MIL-101	-OH	PCMX KET NAP	79.00 80.00 152.00	Electrostatic interaction, Hydrogen-bond interaction	[109]
UiO-66	-OH	MB TC	96.69 37.96	Lewis acid–base interaction, Hydrogen-bond interaction	[110]
UiO-66	-COOH	Sb^3+^	56.49	Electrostatic interaction	[111]
Zr-BTC	-COOH	Sr^2+^	67.50	Electrostatic interaction	[112]
UiO-66-(Zr)	-COOH	Hg^2+^ Ni^2+^	509.80 439.00	Electrostatic interaction	[113]
UiO-66	-F	PFOA	470.00	Hydrophobic interaction	[114]
UiO-66	-F	B	76.30	π–π interaction, Hydrophobic interaction	[115]
SCNU-Z1	-Cl	CrO_4_^2−^ Cr_2_O_7_^2−^ MnO_4_^−^ ReO_4_^−^ MO AO CR MB	126.00 241.00 292.00 318.00 285.00 180.00 585.00 262.00	Hydrophobic interaction	[116]

PFOA: perfluorooctanoic acid; IBF: ibuprofen; ACE: acetaminophen; BPS: bisphenol S; *p*-ASA: arsine acid; MO: methyl orange; MB: methylene blue; MG: malachite green; IC: indigo carmine; BR46: basic Red 46; BB41: basic blue 41; 2,4-d: 2,4-dichlorophenoxyacetic acid; DCF: diclofenac sodium; NAP: naproxen; PCMX: *p*-chloro-m-xylenol; KET: ketoprofen; TC: tetracycline hydrochloride; B: benzene; AO: acid orange A; CR: Congo red.

## Data Availability

Data are contained within the article.
